# A General-Purpose Framework for Microvascular Reconstruction and Quantitative Analysis in Ultrasound Localization Microscopy

**DOI:** 10.34133/bmef.0295

**Published:** 2026-08-03

**Authors:** Peinan Liu, Jie Guo, Xuedi Han, Yuan Zong, Ting Zhang, Ligang Cui, Xuejun Qian

**Affiliations:** ^1^ School of Biomedical Engineering & State Key Laboratory of Advanced Medical Materials and Devices, ShanghaiTech University, Shanghai, China.; ^2^ Eye Institute and Department of Ophthalmology, Eye & ENT Hospital, Fudan University, Shanghai, China.; ^3^ NHC Key Laboratory of Myopia and Related Eye Disease, Chinese Academy of Medical Sciences, Shanghai, China.; ^4^ Shanghai Key Laboratory of Visual Impairment and Restoration, Shanghai, China.; ^5^ Department of Ultrasound, Peking University Third Hospital, Beijing, China.; ^6^ Shanghai Clinical Research and Trial Center, Shanghai, China.

## Abstract

**Objective:** Microvascular alterations are early biomarkers of many diseases, but clinical translation is hindered by the lack of noninvasive deep-tissue imaging tools with capillary resolution and standardized analysis approaches. **Impact Statement:** ULM-based Vascular Biomarker Automated Analysis (U-VBA) provides a standardized framework that bridges microvascular imaging reconstruction and automated biomarker analysis, with feasibility demonstration in animals models in vivo and clinical patient data. **Introduction:** Ultrasound localization microscopy (ULM) can overcome the diffraction limit but lacks generalizable imaging reconstruction and biomarker quantification pipelines. **Methods:** We developed U-VBA, integrating motion correction, cascaded denoising (singular value decomposition, high-pass filtering, and background subtraction), and a panel of 6 vascular biomarkers (density, intervessel distance, diameter, velocity, perfusion, and tortuosity). Performance was validated in various settings, including flow phantoms, chicken chorioallantoic membranes (*n* = 5), rabbit optic nerve injury models (*n* = 4), and a clinical patient cohort with cervical lymph node tumors (*n* = 39). **Results:** U-VBA achieved robust performance in flow phantoms and resolved the finest capillaries down to 16.2 μm in chorioallantoic membrane models. In rabbit eye models, it precisely captures the dynamic vascular changes and establishes the vascular biomarkers during elevated intraocular pressure and recovery. In the clinical cohort, we demonstrated the potential value of U-VBA, which leverages 4 biomarkers (intervessel distance, diameter, perfusion, and tortuosity) to differentiate 3 lymph node conditions, with 85% accuracy in 5-fold cross-validation. **Conclusion:** U-VBA standardizes ULM-based microvascular phenotyping and integrates into routine ultrasound workflows, offering a noninvasive tool for preclinical and clinical vascular biomarker analysis.

## Introduction

The vascular system maintains cellular and organ homeostasis by delivering oxygen and nutrients and removing metabolic waste products [1]. Accumulating evidence indicates that microvascular dysfunction contributes to the development and progression of numerous major diseases, such as cardiovascular [2], metabolic [3], and neurodegenerative diseases [4] and tumors [5], and is thus regarded as an early biomarker in healthcare [6,7]. For instance, coronary microcirculatory dysfunction can lead to myocardial ischemic syndromes [6], systemic microcirculatory abnormalities are related to chronic kidney disease [8], and vascular phenotypes can help diagnose disease across different cancer types [9].

The conventional techniques for microvascular biomarker quantification include the use of immunological methods [10], photoplethysmography [11], and biopsy [12]. However, these approaches cannot directly characterize changes in vascular structure and function, such as vascular density, flow velocity, and perfusion. In addition, the reliability and generalizability of these biomarkers over time, across studies, and disease contexts remain unsolved. Therefore, a standard framework for vascular biomarker quantification that can be used for early disease diagnosis, risk stratification, and dynamic assessment of treatment response is desired.

Due to their high spatiotemporal resolution, imaging-based methods that can resolve microvascular architecture and hemodynamics in deep tissues attract tremendous attention in vascular biomarker quantification. However, existing imaging approaches face an inherent trade-off between spatial resolution and penetration depth. For instance, computed tomography angiography and magnetic resonance angiography provide sufficient penetration depth to fully reconstruct the vascular network, but their millimeter-level spatial resolution cannot visualize important capillary-scale vessels. By contrast, optical techniques such as optical coherence tomography angiography achieve microscale resolution, but their application is restricted to superficial tissues with semiquantitative flow metrics rather than absolute velocities [13].

Ultrasound imaging, recently advanced by plane-wave imaging-based ultrasound localization microscopy (ULM), which enables superresolved microvascular mapping with micron-scale resolution at tissue depths of several centimeters [14,15], opens a new window for precise quantitative measurements of various vascular parameters. The ULM technique has been successfully implemented for many preclinical and clinical studies across the brain [16], heart [17], kidney [18], and eye [19]. However, the potential clinical translation of ULM is still limited by the lack of standardized acquisition/reconstruction protocols and biomarker extraction framework for microvascular assessment [20]. For instance, our previous studies [19,21] demonstrated the potential of ULM in ocular imaging but lacked a standardized acquisition and analysis pipeline, and only 2 biomarkers were examined without longitudinal follow-up. Similarly, in terms of lymph node assessment, Zhu et al. [22] demonstrated that ULM-derived metrics could discriminate metastatic from benign lymph nodes in 10 patients, using local flow direction irregularity. While pioneering, that work was limited to a binary classification in a small cohort and did not explore a broader biomarker panel.

To overcome the limitations of existing research and improve its potential for clinical translation, we developed an integrated framework termed ULM-based Vascular Biomarker Automated Analysis (U-VBA). Such a framework standardizes ULM acquisition, preprocessing, and imaging reconstruction, enabling automated vasculature-based biomarker analysis. We comprehensively evaluated the performance of U-VBA on flow phantoms and chorioallantoic membranes (CAMs) in vitro. U-VBA extracts comprehensive indices of 6 complementary quantitative biomarkers, namely, vascular density, maximum intervascular distance, flow velocity, vessel diameter, perfusion, and tortuosity, simultaneously capturing morphological and hemodynamic properties. Longitudinal in vivo monitoring of optic nerve injury progression in rabbit models confirmed its ability to track dynamic vascular remodeling. Furthermore, artificial intelligence (AI) models were used to distinguish 3 pathological lymph node states (benign, lymphoma, and metastatic carcinoma) in a clinical patient cohort with cervical lymph node tumors (*n* = 39). Our results consistently demonstrated high precision and robustness in algorithm performance and highlighted the potential of the proposed biomarkers for clinical diagnostic applications.

## Results

**Fig. 1.**
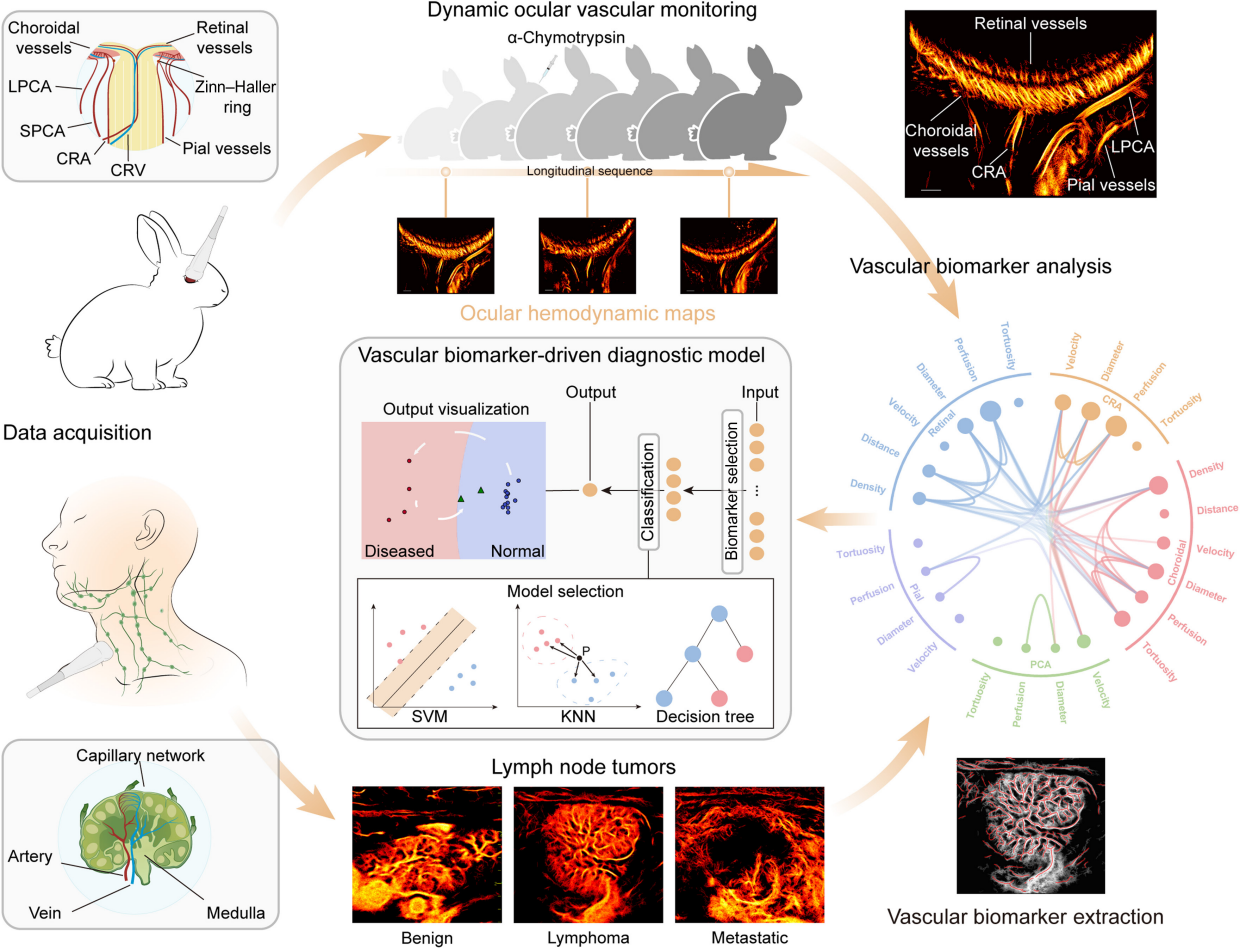
Schematic overview and study design of the ULM-based Vascular Biomarker Automated Analysis (U-VBA) framework. The backbone of this framework is detailed in Fig. 2. A preclinical rabbit model undergoing longitudinal ocular vascular monitoring before and after induced optic nerve injury (upper panel) and a clinical cohort including patients with cervical lymph nodes of different pathological types (bottom panel) were used to demonstrate the generalizability of the framework. The experimental procedures for both studies include ultrasound data acquisition and reconstruction, extraction and analysis of vascular biomarkers, and construction of diagnostic models. The hierarchical edge-bundling diagram (right) illustrates relationships among ocular biomarkers. Each circular leaf node represents a specific biomarker measured in a particular vascular structure. Node size is proportional to the magnitude of the statistically significant difference observed for that biomarker in this study; larger nodes indicate a greater statistically significant difference. Connections are drawn between biomarkers with a Pearson correlation coefficient |*r*| > 0.6. ULM, ultrasound localization microscopy.

**Fig. 2.**
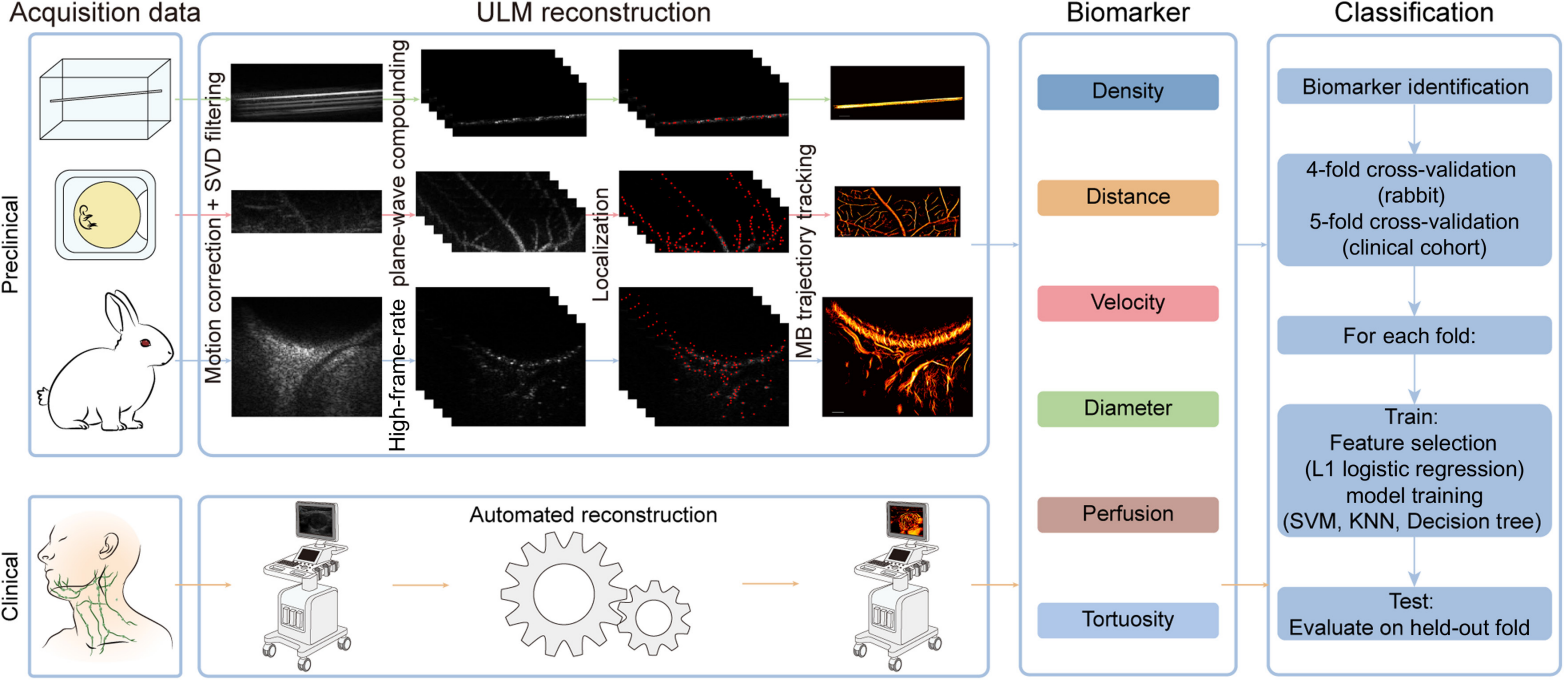
Ultrasound localization microscopy (ULM) image reconstruction and data analysis pipeline. The pipeline consists of 4 stages: data acquisition, ULM image reconstruction, vascular biomarker extraction and analysis, and development of a disease classification model. Data acquisition comprised preclinical and clinical studies. Preclinical data included (a) a flow phantom, (b) a chick embryo model, and (c) a rabbit model of optic nerve injury; the clinical cohort consisted of patients with neck lymph nodes of different pathological types. Preclinical ULM images were reconstructed with a robust processing pipeline optimized for ocular vasculature, incorporating standard steps of motion correction, denoising, microbubble localization, and trajectory tracking. Clinical ULM images were generated directly by the scanner’s vendor-provided reconstruction algorithm. The flow phantom and chick embryo experiments were used solely to validate and assess algorithm performance; only in vivo datasets (rabbit model) and patient cohorts proceeded to downstream biomarker extraction and diagnostic classification.

As shown in Fig. 1, the U-VBA workflow encompasses standardized data acquisition, superresolution image reconstruction, quantitative biomarker extraction, and AI-based automated disease classification. The backbone of this framework is a robust ULM reconstruction pipeline including motion correction, microbubble (MB) signal separation, precise localization, and trajectory tracking (Fig. 2), which serves as the foundation for downstream biomarker analysis.

### Robust vascular biomarker quantification via U-VBA

Before conducting in vivo experiments, we systematically evaluated the accuracy of U-VBA reconstruction images and the reliability of biomarker quantification through in vitro experiments, including flow phantom experiments and CAM experiments, to establish the foundation for subsequent in vivo validations.

In the flow phantom experiments, a 300-μm-inner-diameter silicone tube was placed in a water tank, and the flow velocity was precisely controlled by a syringe pump, set at 10, 20, 40, 60, 80, 100, and 120 mm/s, respectively. Based on our U-VBA reconstruction, the calculated average velocities were 9.77, 20.23, 41.54, 59.23, 80.44, 101.13, and 115.2 mm/s, respectively (Fig. S1a to h). Across the setting range (10 to 120 mm/s), the maximum relative error between the ground truth and the U-VBA-reconstructed velocity was less than 4%, demonstrating the accuracy of U-VBA in velocity quantification. In addition, the U-VBA-measured tube inner diameter was 295 μm (Fig. S1i), with a relative error of approximately 1.67% from the ground truth value of 300 μm, demonstrating the reliability of U-VBA in reconstructing image structures.

To further evaluate U-VBA’s ability to reconstruct complex vascular networks and its spatial resolution, we used a model of the vascular-rich, transparent CAM and compared it to images obtained from an optical microscope as a gold standard. The U-VBA-reconstructed vasculature (Fig. S2a) showed good spatial correspondence with the optical reference image (Fig. S2b), demonstrating U-VBA’s fidelity in capturing vascular morphology. The diameters of the smallest and largest vessels measured in the flow velocity map generated by U-VBA were 16.2 and 233.5 μm, respectively. These errors corresponded to measured diameters of 17.6 and 237.4 μm, representing 7.95% and 1.64% errors, respectively. These results confirm U-VBA’s ability to capture morphological information from microvessels to larger vessels accurately.

### The performance of U-VBA in animal models

We first performed experiments on the same rabbit eye to assess the performance of U-VBA in reconstructing in vivo vasculature and experimental reproducibility. We compared U-VBA reconstructions with those from the benchmark algorithm, Performance Assessment of Localization Algorithms (PALA) [15]. For a fair comparison, we first optimized PALA’s parameters (see Materials and Methods). Under these optimized settings, PALA was unable to reconstruct retinal vessels (Fig. S3). When PALA was applied without any bubble-per-frame limit, it did visualize retinal vasculature; however, the reconstructions contained substantial background noise from spurious localizations in avascular areas, which obscured fine vascular details and prevented reliable biomarker quantification (Fig. S4). In contrast, U-VBA consistently generated clean vascular maps with low background signals, enabling the clear distinction of retinal and choroidal microvessels. These results demonstrate that U-VBA has a superior capability in resolving finer vascular details, particularly in reconstructing microvessels such as retinal and choroidal vessels, which form the basis for the subsequent multiparametric analysis.

In a separate experiment to assess test–retest reproducibility, the same rabbit eye was imaged on 2 different days. Although the imaging planes were not identical (Fig. S5) due to operator-dependent cross-sectional ultrasound imaging, we quantified the same major vessels (e.g., central retinal artery [CRA], lateral posterior ciliary artery [LPCA], and pial vessels) to ensure consistency. For microvessels such as retinal and choroidal vessels, we used average values to represent the overall status. We extracted and compared biomarkers from the ULM images. The average difference between the 2 extractions was 3.44%, with a maximum difference of approximately 10% (Table S1). The intraclass correlation coefficient was 0.981, demonstrating excellent reproducibility of the U-VBA framework.

Dynamic changes in the vascular system during disease progression and treatment are crucial for understanding disease mechanisms, diagnosis, and prognosis. To evaluate the capability of U-VBA for dynamically monitoring temporal changes in vascular status, we applied it to a cohort of rabbits with induced ocular hypertension. To assess the progression and recovery of vascular changes, we performed longitudinal imaging at 3 specific phases: normal at phase 1, progression at phase 2, and recovery at phase 3 (see Materials and Methods). The experiment involved 4 rabbits, each modeled to have one eye with induced glaucoma and one normal eye, allowing for direct comparison of vascular changes.

**Fig. 3.**
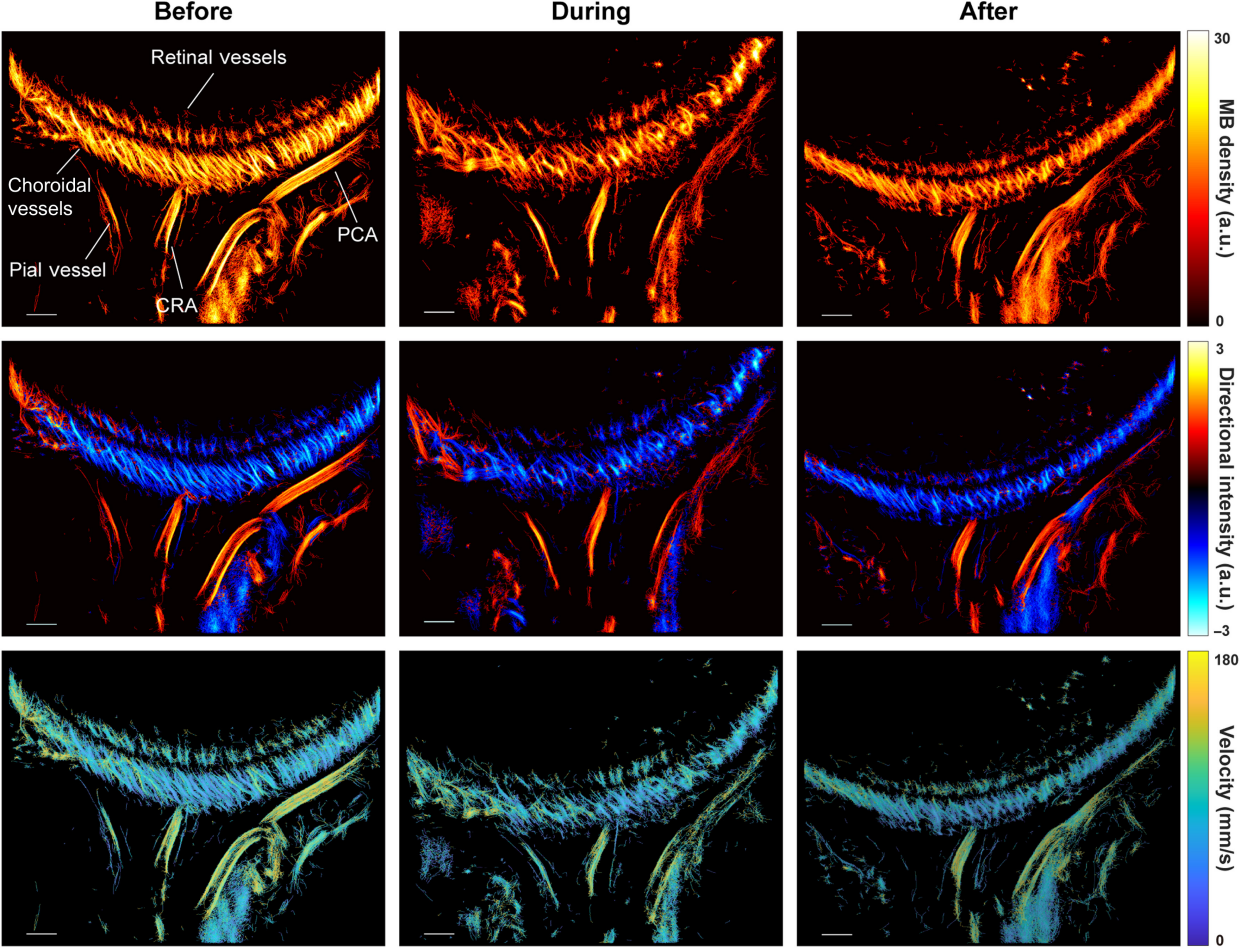
Hemodynamic maps of the rabbit optic nerve injury model reconstructed by ultrasound localization microscopy (ULM) at multiple time points. The images show (from left to right) the baseline, 4 weeks post-induction, and 5 weeks post-induction. The rows display blood flow density, directional flow density, and flow velocity maps, respectively. Scale bar, 1 mm.

U-VBA successfully reconstructed high-fidelity hemodynamic maps of the rabbit eye at each time point, clearly distinguishing key ocular vessels including the CRA, the LPCA, pial vessels, choroidal vessels, and retinal vessels (Fig. 3) [23]. This reliable separation of retinal and choroidal vasculature, which was challenging in previous ULM studies of the eye [19], provided the necessary image quality for the quantitative biomarker analysis that follows. Qualitative assessment revealed that the vessel density in phase 2 was substantially lower than that in phase 1, with a partial recovery observed in phase 3, demonstrating the ability of U-VBA to visualize dynamic vascular alterations. In addition, U-VBA reliably quantified high flow velocities (>100 mm/s) in ocular vessels such as the CRA and LPCA, outperforming previous ULM studies in rabbit eyes, which were limited to velocities below 10 mm/s [19,24].

**Fig. 4.**
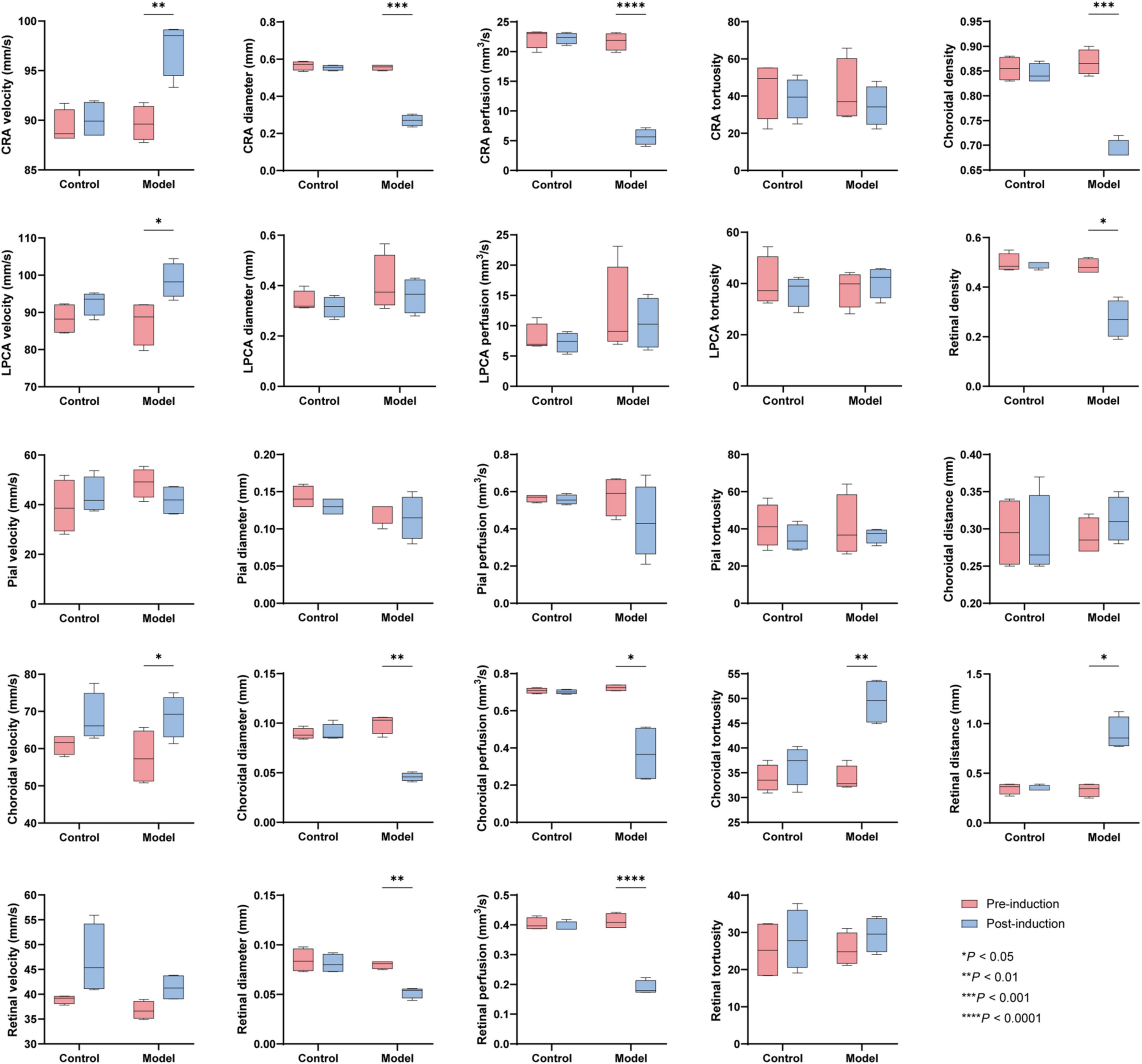
Comparison of vascular biomarkers in the main ocular vessels before and after induction of optic nerve injury in the rabbit model. Statistical analyses were performed using paired *t* tests for normal data or Wilcoxon signed-rank tests for nonnormal data, with Holm–Šidák correction for multiple comparisons. Significance levels: *P < 0.05; **P < 0.01; ***P < 0.001; ****P < 0.0001.

U-VBA quantified multiple biomarkers, including vessel density, maximum intervascular distance, average blood flow velocity, vessel diameter, blood perfusion, and vascular tortuosity, across 5 ocular vascular types. Vessel density and maximum intervascular distance were quantified in the choroidal and retinal vasculature due to their structural richness. Paired *t* tests and Wilcoxon signed-rank tests (with Holm–Šidák multiple-comparison correction) were then used to assess changes in biomarkers between phases 1 and 2 to identify those that could effectively detect optic nerve damage (Fig. 4 and Fig. S6a). Optic nerve injury induced significant hemodynamic changes between phases 1 and 2. The CRA showed an increased flow velocity (*P* < 0.01), a decreased diameter (*P* < 0.001), and reduced perfusion (*P* < 0.0001). Concurrently, the LPCA also exhibited an increased flow velocity (*P* < 0.05). Choroidal vessels demonstrated a decreased vascular density (*P* < 0.001), an increased flow velocity (*P* < 0.05), a decreased diameter (*P* < 0.01), reduced perfusion (*P* < 0.05), and increased tortuosity (*P* < 0.01). Retinal vessels showed a decreased vascular density (*P* < 0.05), an increased maximum intervascular distance (*P* < 0.05), a decreased diameter (*P* < 0.01), and reduced perfusion (*P* < 0.0001). Overall, the analysis revealed that 13 of the 24 biomarkers analyzed showed statistically significant changes after injury. In contrast, no significant changes were observed in the control group, highlighting the potential of U-VBA for detecting injury-related vascular dysfunction.

To validate the biomarker alterations detected by U-VBA against pathological findings, we euthanized the rabbits and collected their eyes for subsequent hematoxylin–eosin staining. The analysis revealed significant structural damage in the experimental eyes compared to the controls (Fig. S7). Specifically, the experimental eyes exhibited severe loss of retinal ganglion cells, thinning of the retinal nerve fiber layer, and a moderate reduction in cell number within the inner nuclear layer. These pathological features hallmark glaucomatous optic neuropathy [25], confirming that the hemodynamic impairment captured by U-VBA was associated with neural damage.

In phase 3, biomarker levels in the experimental group began to converge toward the values observed in phase 1, with some returning to normal levels, indicating the ability of the vascular system to adapt and recover after normalization of intraocular pressure (IOP). Furthermore, 11 of the 24 biomarkers showed no significant differences, likely due to limited statistical power from the small sample size rather than a lack of association with optic nerve damage. These findings confirm the ability of U-VBA to detect dynamically changing vessels and highlight its potential as a tool for monitoring vascular pathophysiology.

To further explore the interrelationships between the biomarkers, we calculated a Pearson correlation matrix (Fig. S6b) and visualized the correlation structure using a hierarchical edge-bundling plot (Fig. 1), illustrating the biomarker correlation network and its association with optic nerve damage status.

### The performance of U-VBA in patients

**Fig. 5.**
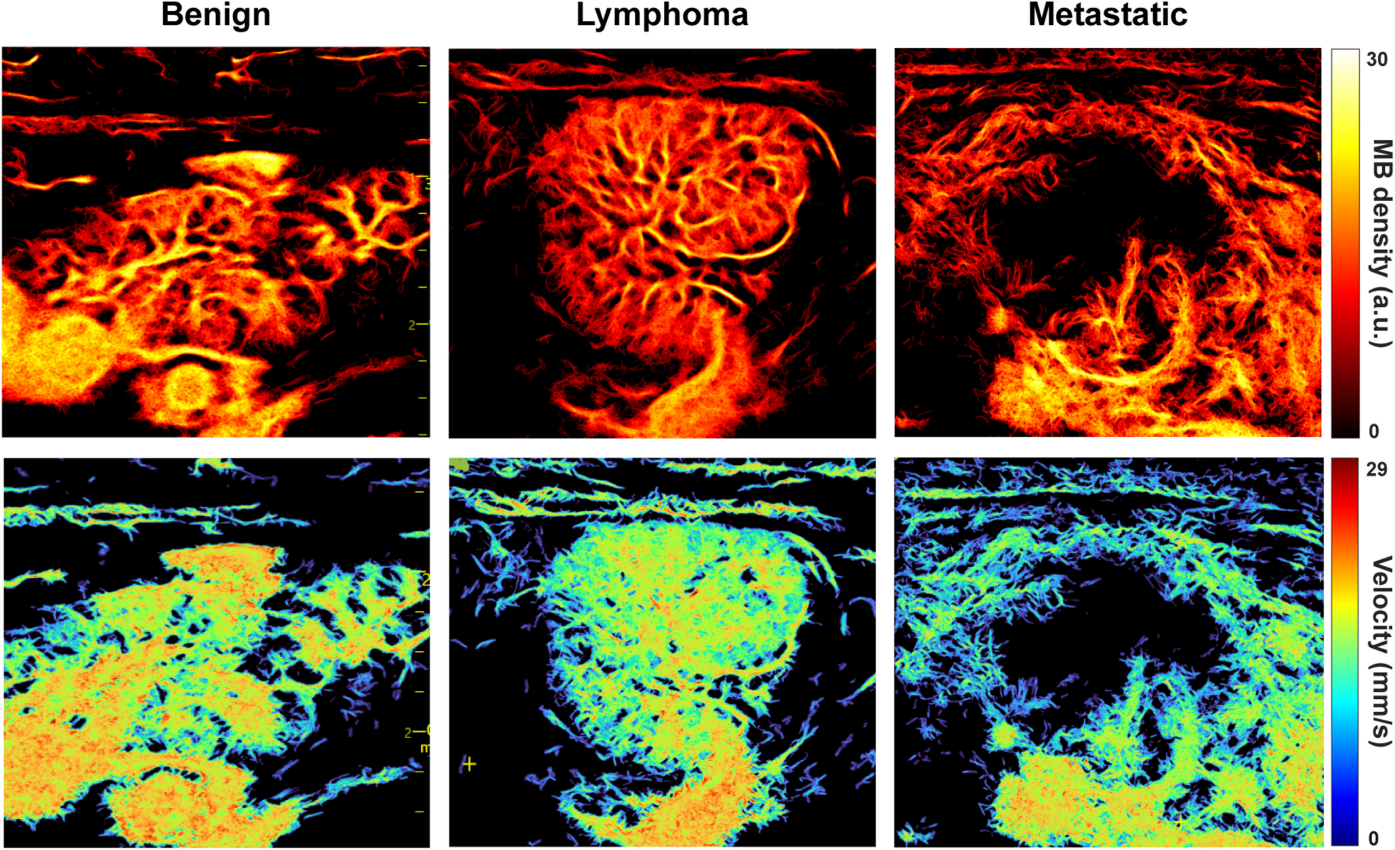
Ultrasound localization microscopy (ULM)-derived hemodynamic maps of lymph nodes with different pathologies in patient cohorts. Columns (left to right) show representative benign, lymphoma, and metastatic carcinoma nodes. Rows (top to bottom) display blood flow density and flow velocity maps. Scale bar: 1 mm.

Following validation in preclinical studies, we applied U-VBA to a cohort of patients with cervical lymph node disease to evaluate its integration into the standard clinical workflow and its ability to effectively differentiate between different disease types noninvasively. A total of 39 patients (12 benign lymph nodes, 15 lymphomas, and 12 metastatic carcinomas) were collected. All imaging was acquired by an expert sonographer using a commercial ultrasound system (ULTIMUS 9E, VINNO Technology, Suzhou, China), followed by its built-in ULM processing pipeline before being analyzed using our U-VBA framework (Fig. 5).

**Fig. 6.**
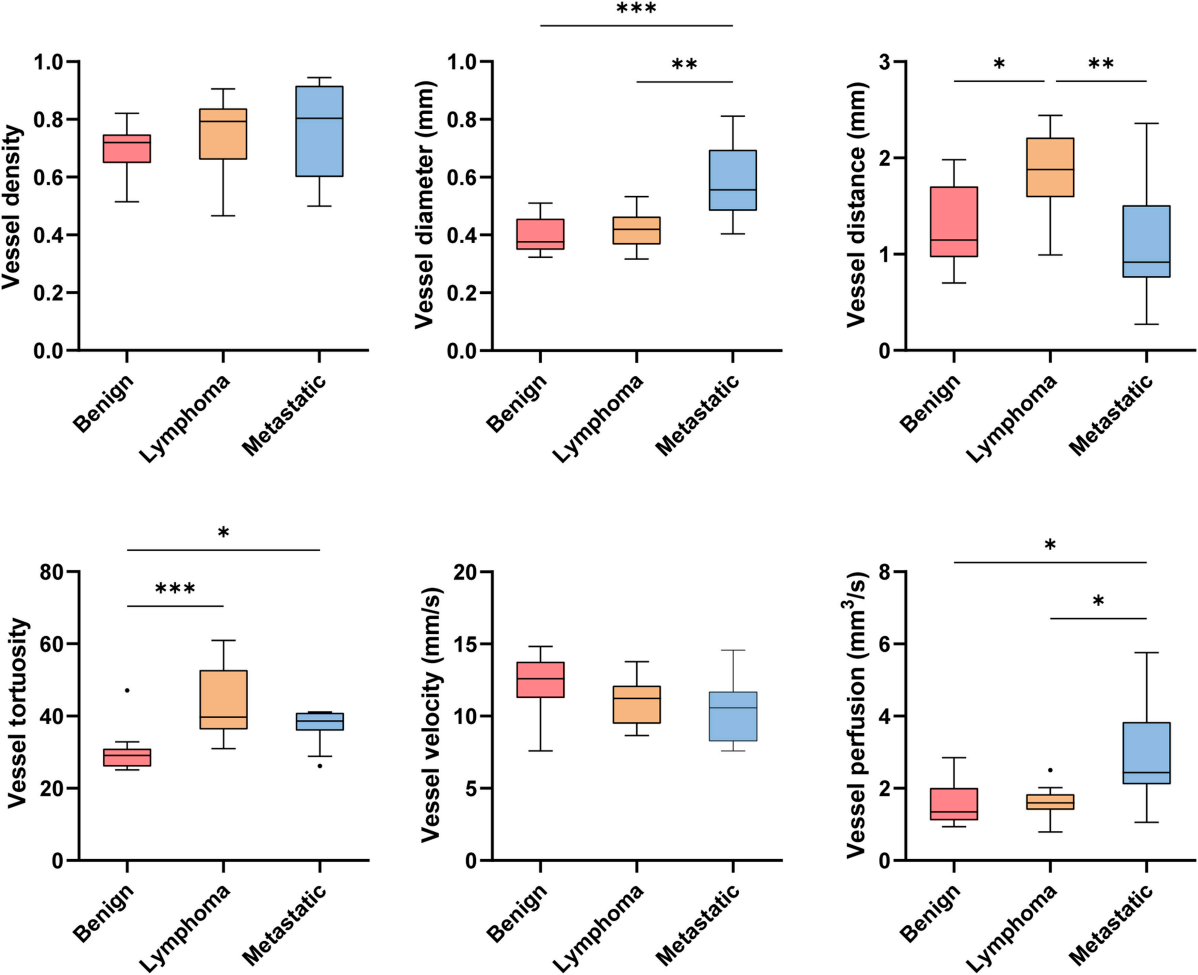
Comparison of vascular biomarkers across lymph node pathologies in patients. For each biomarker, a one-way analysis of variance (ANOVA) was performed; pairwise differences were assessed with Tukey’s post hoc multiple comparisons test. Significance: *P < 0.05; **P < 0.01; ***P < 0.001.

Specifically, we examined the entire vascular population rather than specific vascular types to ensure a comprehensive assessment of vascular characteristics. We analyzed 6 quantified biomarkers: vessel density, maximum intervascular distance, mean blood flow velocity, vessel diameter, blood perfusion, and vascular tortuosity. Of these, 4 biomarkers, namely, vessel diameter, maximum intervascular distance, blood perfusion, and tortuosity, showed significant differences (*P* < 0.05) among the 3 disease types when analyzed using the one-way analysis of variance (ANOVA). The results showed significant specificity between the different tumor types for vessel diameter, maximum intervascular distance, blood perfusion, and tortuosity (Fig. 6).

While no single biomarker perfectly differentiated the 3 tumor types, different biomarkers, such as vessel diameter and tortuosity, exhibited varying pathological specificity. For example, vessel diameter distinguishes benign lymph nodes from metastatic cancer (*P* < 0.001) and lymphoma from metastatic cancer (*P* < 0.01), but it does not distinguish between benign lymph nodes and lymphoma. Conversely, tortuosity effectively distinguishes benign lymph nodes from lymphoma (*P* < 0.001) and benign lymph nodes from metastatic cancer (*P* < 0.05), but it does not distinguish between lymphoma and metastatic cancer. Therefore, these 2 biomarkers provide complementary discriminatory capabilities. This multifaceted biomarker profile demonstrates that U-VBA can noninvasively quantify multiple vascular biomarkers, providing a promising foundation for differentiating between benign lymph nodes, lymphomas, and metastatic carcinomas.

### Multidimensional biomarkers enable automated disease prediction

To validate the effectiveness of the multidimensional biomarkers that U-VBA provides for automated disease diagnostic classification, we developed and evaluated 3 AI models (a support vector machine [SVM], a *k*-nearest neighbor [KNN], and a decision tree) using 2 distinct datasets (a rabbit optic nerve injury dataset and a patient cervical lymph node tumor dataset).

For the rabbit optic nerve injury dataset, 13 biomarkers (statistical significance between phases 1 and 2) were fed into the AI model. The training dataset consisted of 16 samples: longitudinal scans of both eyes from 4 modeling rabbits during phases 1 and 2. All phase 1 scans (*n* = 8 eyes) were normal. In phase 2, scans of the experimental eye (*n* = 4) were labeled as diseased, while the contralateral control eye (*n* = 4) remained normal. Therefore, the training dataset contained 12 normal samples and 4 diseased samples. Each classifier was evaluated using a 4-fold cross-validation algorithm, maintaining a 3:1 ratio of normal to abnormal samples. All 3 classifiers performed well in identifying vascular status, achieving 100% accuracy and an F1 score of 1.0 (Fig. S6c).

We further evaluated the trained classifiers on an independent test set consisting of phase 3 data from 2 eyes in the experimental group and phase 1 data from 8 eyes of 4 rabbits, in which modeling failed. All classifiers correctly classified each control eye as normal. In phase 3, biomarkers exhibit a transitional state between phases 1 and 2. When the trained classifiers were applied to the phase 3 data, both eyes were classified as normal. In the principal component analysis (PCA)-reduced space, the data points in phase 3 lie near the decision boundary, tending toward the normal cluster but still separable from it, suggesting a transitional state (Fig. 1). While phase 3 represents a state of partial recovery distinct from normal physiology, the lack of a dedicated transitional disease state label precludes precise assessment. These findings demonstrate the potential of biomarkers to track disease progression.

For the patient cervical lymph node tumor dataset, we used biomarkers that showed significant differences between any 2 disease states as model inputs. Five-fold cross-validation was used to evaluate model performance. Stratified sampling was used to partition the dataset into training and test sets in an 8:2 ratio, ensuring consistent proportions of disease classes. We trained the SVM, KNN, and decision tree models and evaluated each model using stratified 5-fold cross-validation. The SVM model achieved the best overall performance, with an average accuracy of 0.85 (±0.05), substantially outperforming both the KNN (0.80 ± 0.10) and the decision tree (0.75 ± 0.16). These results demonstrate the potential of U-VBA-extracted biomarkers for effectively classifying multiple disease types, highlighting their applicability in AI diagnostic models.

## Discussion

Microvascular dysfunction is an early biomarker for a variety of diseases. However, there currently lack noninvasive and effective imaging tools to visualize deep tissues at a capillary resolution, as well as standardized, multidimensional biomarker systems for systematically characterizing vascular phenotype [26]. In this study, we developed and validated U-VBA, an integrated framework that enables standardized ultrasound microvascular imaging, quantitative vascular phenotyping, and intelligent diagnostics, demonstrating its potential clinical translation for longitudinal disease monitoring and prediction.

U-VBA demonstrated excellent quantitative accuracy in flow phantom, CAM (*n* = 5), and animal models (*n* = 4), reconstructing complex vascular networks and visualizing capillaries as fine as 16.2 μm while assessing vascular phenotypes across multiple dimensions. U-VBA also outperformed the PALA benchmark in visualizing fine vascular details, particularly retinal and choroidal microvasculature. Furthermore, U-VBA provided highly reproducible vascular biomarker estimates, enabling the identification of differential damage across ocular vessels in the setting of optic nerve injury models in vivo. In the rabbit optic neuropathy model, U-VBA captured a series of structural and functional changes in blood vessels, including decreased density, diameter, and perfusion. These changes partially recover after normalization of IOP, showing vascular plasticity and potential for monitoring disease progression. In addition, biomarker alterations were primarily observed in vessels supplying the optic nerve head, including the CRA, retinal vessels, and choroidal vessels. This spatial heterogeneity highlights the ability of U-VBA to localize lesions precisely. Notably, as the only organ where microvasculature can be directly observed, the eye provides a unique biological window for monitoring systemic health [27]. Recent advances in deep-learning-based AI models [28–31] have shown tremendous potential for medical image analysis and the assessment of associated biomarkers. U-VBA can expand this paradigm by providing standardized, multidimensional biomarker profiles, and it combines these with AI diagnostic models, which potentially enable the monitoring of ocular and even systemic diseases.

In addition to previous preclinical animal model demonstrations, U-VBA has demonstrated strong discriminatory ability in clinical patient studies. Due to the complexity of the disease characteristics [32,33], histopathology remains the gold standard in clinical practice; however, biopsies are invasive and time-consuming [34]. U-VBA addresses this problem by noninvasively constructing a comprehensive, multidimensional biomarker profile that integrates complementary vascular features. In patient cohorts (*n* = 39) with cervical lymph node tumors in this study, U-VBA can identify differences in diameter, vessel spacing, vascular tortuosity, and blood perfusion between benign lymph nodes, lymphomas, and metastatic carcinomas. Integrating these features into AI models enables accurate multiclass diagnosis, providing a noninvasive alternative to invasive biopsies and histopathology. Importantly, U-VBA can be seamlessly integrated into existing standard ultrasound workflows, enabling microvascular imaging, biomarker extraction and analysis, and assisting scientists and physicians in disease diagnosis.

This study has several limitations. First, given the small sample size of the rabbit model, the machine learning results are presented solely as a proof-of-concept demonstration that ULM-derived vascular biomarkers carry biologically meaningful signals associated with disease states, and larger-scale validation is required. Second, current ultrasound imaging is limited to 2-dimensional (2D) imaging, which can introduce bias in the quantification of vascular biomarkers and fail to capture important 3-dimensional (3D) spatial information, such as vascular network topology [35]. Third, the ULM imaging still requires the injection of contrast agents, making it not fully noninvasive. We are currently working to extend U-VBA to 3D ultrasound imaging and broaden the biomarker profile to develop a comprehensive vascular monitoring framework. We are also employing contrast-free imaging algorithms to achieve noninvasive imaging, reduce computational and reagent costs, and accelerate the clinical translation of U-VBA.

In summary, our study established a U-VBA framework based on ULM technology for extracting and automatically analyzing vascular biomarkers. Its potential wide clinical applications range from monitoring vascular changes to assessing disease progression, or to treatment efficacy, all of which can be beneficial to standard clinical workflows for supporting multiple disease predictions.

## Materials and Methods

### Flow phantom experiment

To assess the robustness of ULM in quantifying flow velocity, a flow phantom experiment was conducted. A silicone tube, with an inner diameter of 300 μm and an outer diameter of 800 μm, was straightened and secured within a water tank. One end of the tube was connected to a syringe pump (LSP01-2Y, Rongbai, China) to regulate the flow rate. SonoVue MBs (Bracco Diagnostics Inc., Monroe Township, NJ, USA) were diluted with saline to approximately 1/700 times to serve as the perfusion solution. Flow velocities were set to 10, 20, 40, 60, 80, 100, and 120 mm/s. All imaging was performed with a Verasonics Vantage ultrasound system (Verasonics Inc., Kirkland, WA, USA) and an L22-14vX linear array probe. Continuous ultrasound data acquisition was performed with a center frequency of 15.625 MHz and a transmit voltage of 10 V. For both the flow phantom and animal experimental setups, 7-angle compounding plane-wave imaging was implemented with an angle step size of 2° and a compounded frame rate of 1,000 Hz. A total of 16 s of radio-frequency (RF) data were collected. The acquired RF data were beamformed into in-phase and quadrature (IQ) data using a delay-and-sum (DAS) algorithm.

### CAM experiment

To evaluate the imaging accuracy of ULM and the range of vessel diameters that can be measured, the CAM was used to compare the differences between the vascular reconstructions of ULM and the images taken with an optical camera. The CAM preparation and ultrasound acquisition setup were followed as previously described [36,37] to obtain ultrasound data of the CAM. In this experiment, we used 5 CAMs in total. CAM optical imaging was performed using an optical camera. The scale of the optical photograph was determined by comparing the length of the ultrasound probe in the image to its actual physical length. Continuous ultrasound data acquisition was performed with a center frequency of 15.625 MHz and a transmit voltage of 10 V. Plane-wave compounding imaging was performed using 12 transmission angles with a 2° angular increment and a compounded frame rate of 500 Hz. A total of 34 s of RF data were collected. The acquired RF data were beamformed into IQ data using a DAS algorithm.

### Rabbit optic neuropathy model

A total of 8 female New Zealand White rabbits (~2 kg) were utilized. One eye per rabbit was randomly selected as the experimental eye, with the contralateral eye serving as an internal control. Under 2% isoflurane general anesthesia, 0.15 ml of α-chymotrypsin solution with a concentration of 500 U/ml was injected into the anterior chamber of the experimental eye to induce optic nerve damage. The control eye received an equivalent volume of saline using the same procedure [38]. IOP was monitored 3 times per week using a tonometer (FA800vet, FuAn, China). Eyes sustaining an elevated IOP for 4 weeks were defined as glaucomatous. Ultimately, a total of 2 left eyes and 2 right eyes were considered to have developed glaucoma.

### Rabbit eye imaging and processing

Before the experiment, rabbits were fasted for at least 12 h and deprived of water for 4 h. In the experiment, the rabbits were initially anesthetized in an induction chamber with 5% isoflurane. Subsequently, the rabbits were placed on a heating pad to maintain body temperature throughout the procedure. To sustain anesthesia, a 1:1 mixture of Zoletil 50 and xylazine hydrochloride injection was prepared and administered at a dose of 1 ml/kg via intramuscular injection into the lateral hind-limb muscle using a 1-ml syringe. To suppress reflexive ocular movements, topical ocular anesthesia was achieved by instilling 2 drops of 0.4% oxybuprocaine hydrochloride ophthalmic solution into the conjunctival sac of each eye.

In this longitudinal study, a standardized acquisition protocol was employed to ensure that the anterior–posterior alignment of the acquired images was as consistent as possible across time points. The ultrasound probe was positioned along the canthal–external auditory meatal line, angled at approximately 75° relative to the sagittal plane of the rabbit (Fig. 1). At each subsequent imaging session, the probe position was adjusted to maintain consistency with the initial acquisition position. Continuous ultrasound data acquisition was performed with a center frequency of 15.625 MHz and a transmit voltage of 10 V. Seven-angle compounding plane-wave imaging was implemented with an angle step size of 2°, and a total of 22 s of RF data were collected. The acquired RF data were beamformed into IQ data using a DAS algorithm.

Before proceeding with modeling, all rabbit eyes underwent a single data acquisition to establish a baseline for comparison with subsequent measurements. For rabbits in which glaucoma was successfully induced, we performed a second data acquisition 4 weeks after injection. Additionally, to assess the progression of changes over time, the experimental eyes of 2 randomly selected experimental rabbits underwent an additional imaging session 5 weeks postinjection. We defined the pre-induction time point as phase 1, the 4-week postinjection time point as phase 2, and the 5-week postinjection time point as phase 3.

### ULM image processing

The data processing workflow is illustrated in Fig. 2. The ULM process includes data preprocessing, MB positioning, MB trajectory tracking, and the accumulation of trajectories to generate a hemodynamic map. The acquired IQ data were processed to generate B-mode images. The first frame was designated as the reference image for motion correction. Geometric transformations based on 2D phase correlation estimation [39] were applied to align subsequent frames with the reference, correcting for motion artifacts caused by physiological movements such as respiration or cardiac activity. Next, singular value decomposition (SVD) filtering with an adaptive cutoff was applied to the IQ data to suppress tissue signals [40]. The entire imaging region (200 × 130 pixels) was processed as a single block, and the temporal window length was 800 frames. For each pixel, the adaptive cutoff was determined as the first inflection point of the singular value curve, which naturally separates the tissue and blood signal subspaces. For the rabbit eye dataset, a 50-Hz high-pass filter was additionally employed to remove residual tissue signals. A region of interest (ROI) was manually defined within the vitreous body, and the standard deviation of the signal intensity within this ROI was calculated and subtracted from the entire dataset to remove noise. To enhance MB pairing accuracy, the dataset was separated into 2 subsets based on the direction of MB motion. Specifically, a 3D fast Fourier transform was applied to the IQ data along the 2 spatial axes and the temporal axis. In the resulting frequency domain, the sign of the temporal frequency directly encodes the axial direction of motion: positive temporal frequencies correspond to motion toward the transducer, and negative temporal frequencies to motion away from the transducer [37]. MB localization was performed using a Gaussian function of the point spread function [41], with an assumed point spread function size of 3 × 3 pixels. This kernel size was selected because, in preliminary tests on rabbit eye data, the 3 × 3 kernel yielded higher localization accuracy compared with 5 × 5 and 7 × 7 pixel kernels. The Kuhn–Munkres algorithm was utilized to pair MBs into trajectories [42]. Only trajectories spanning at least 10 consecutive frames were retained to ensure reliability. Finally, a flow intensity map was reconstructed by accumulating MB trajectories and interpolating the data by a factor of 7. The velocity of each MB was derived from positional changes across adjacent frames, using the known frame rate. A velocity map was then generated by averaging velocities from multiple trajectories.

To verify the performance of our ULM reconstruction algorithm, we also reconstructed the same data using the ULM benchmark algorithm PALA [15]. We optimized its key parameters to achieve the best possible performance of PALA. The SVD clutter filter cutoff was set to 30, and the localization method was set to radial symmetry. For the maximum number of bubbles per frame, we compared 2 settings: no limit and a limit of 200. The limit of 200 was chosen based on the observation that, in our data, U-VBA typically localized slightly more than 100 but fewer than 200 bubbles per frame; thus, 200 represents a generous upper bound that accommodates legitimate MB signals while excluding excessive noise. All other PALA parameters were kept at their default values.

### Imaging protocol for clinical cervical lymph node tumors

A total of 39 patients with enlarged superficial lymph nodes were recruited in this prospective study between October 2024 and September 2025. All patients had a pathological diagnosis. Written informed consent was obtained from all enrolled patients. In addition to routine ultrasound examination, ULM was also performed using the U5-15 probe of the ULTIMUS 9E instrument (VINNO Technology, Suzhou, China). The patient received 0.2 ml of SonaZoid MB suspension via peripheral intravenous injection to assess the real-time microvascular distribution of lymph nodes. A total of 30 s of ultrasound data were collected, and the ULM image was directly reconstructed by ULTIMUS 9E.

### Definition and measurement of biomarkers

Six hemodynamic biomarkers were quantified, namely, vascular density, maximum intervascular distance, flow velocity, vessel diameter, perfusion, and tortuosity. Vascular density was defined as the proportion of vessel area relative to the total area of the selected region [19], expressed as

$$\mathrm{VD}=\frac{A_{v}}{A_{\mathrm{Roi}}}$$ (1)

where VD is the vascular density, $A_{v}$ is the area of vessels, and $A_{\mathrm{Roi}}$ is the total area of the selected region.

The maximum intervascular distance was defined as the greatest distance between adjacent vessels, given by

$$\mathrm{MID}=\max\left\{\min\left[d\left(\partial V_{i},\;\partial V_{j}\right)\right],\forall i,\;j\right\}$$ (2)

where MID is the maximum intervascular distance, d is the Euclidean distance between the centers of adjacent vessels i and j, and ∂V denotes the vessel boundary.

The flow velocity was determined from a velocity profile extracted along a line perpendicular to the vessel axis. The maximum velocity was defined as the peak value of this profile. Assuming that the flow is laminar and follows Poiseuille’s law, the average velocity was approximated as half of the maximum velocity. This average velocity of the blood vessel was taken as the representative flow velocity, calculated as

$$V_{\text{flow}}=V_{\mathrm{avg}}=\frac{V_{\max}}{2}$$ (3)

where $V_{\mathrm{avg}}$ is the average velocity and $V_{\max}$ is the maximum velocity.

Vessel diameter was estimated as the full width at half maximum (FWHM) of the normalized density profile, denoted as

$$D=\text{FWHM}\left(d\left(x\right)\right)$$ (4)

where $d\left(x\right)$ is the normalized density profile.

Perfusion was calculated as

$$Q=\pi r^{2}V_{\mathrm{avg}}$$ (5)

where *Q* is the perfusion and *r* is the radius.

The tortuosity of the vessel was calculated using the inflection count metric (ICM). The skeleton of the vessel was first extracted using ImageJ. ICM counted the number of inflection points in the centerline of the vessel and multiplied this number (plus 1) times the total path length and divided by the distance between the endpoints [43]. Finally, it was divided by the number of blood vessels to obtain the average tortuosity of the blood vessels. ICM was calculated as

$${\mathrm{ICM}}_{\mathrm{avg}}=\frac{1}{N}\sum_{i=1}^{n}\frac{\left(n_{i}+1\right)L_{i}}{d_{i}}$$ (6)

where N is the total number of blood vessels, $n_{i}$ is the number of inflection points on the centerline of the *i*th vessel, $L_{i}$ is the total path length of the *i*th vessel, and $d_{i}$ is the straight-line distance between the endpoints of the *i*th vessel.

These biomarkers were quantified in major ocular vessels supplying the optic nerve, including the CRA, the LPCA, pial vessels, choroidal vessels, and retinal vessels. Vascular density and maximum intervascular distance were restricted to the retina and choroid.

### Vascular biomarker-driven diagnostic AI model

We trained 2 classification models based on the preclinical animal model dataset and the clinical patient cervical lymph node tumor dataset. For the preclinical animal model dataset, the AI diagnostic model was developed using longitudinal data from 8 eyes of 4 experimental rabbits, with each eye providing pre-induction and post-induction optic nerve damage measurements (16 total samples). Statistically significant biomarkers (*P* < 0.05, Holm–Šidák corrected), as identified in the statistical analysis section, were extracted from these datasets and utilized as input features for model training. To ensure data consistency, all features were standardized, and feature selection was performed using L1-regularized logistic regression. Three machine learning models, including SVM, KNN, and decision tree, were employed to assess the generalizability of these biomarkers. Model training and evaluation were conducted using a 4-fold cross-validation strategy, maintaining a class distribution of 3:1 (normal to abnormal) across both training and testing partitions. Performance was assessed via confusion matrices, and PCA was applied to reduce the feature space to 2 dimensions, enabling visualization of the decision boundaries (Fig. 1).

To avoid data leakage and ensure rigorous model evaluation, all feature standardization, feature selection (L1-regularized logistic regression), and any statistical screening were performed strictly within the training set of each cross-validation fold. Specifically, these procedures were applied only to the training data, and the selected feature subsets were then used to train the model and evaluate on the held-out test fold. This ensures that the test data remained completely unseen during feature selection and model training. The same procedure was applied to both the preclinical animal dataset and the clinical patient dataset.

To further evaluate the sensitivity of the proposed biomarkers in monitoring changes in vascular status, we analyzed an independent validation set consisting of phase 3 data from 2 eyes in the experimental eyes and phase 1 data from 8 eyes of 4 rabbits in which modeling failed. The validation results are illustrated through a 2D PCA plot (Fig. 1).

The clinical patient cervical lymph node tumor dataset contains data from 39 patients, namely, 12 with benign lymph nodes, 15 with lymphoma, and 12 with metastatic carcinoma. Following the same data processing and cross-validation strategy described above, biomarkers that showed significant differences between any 2 disease types within each training fold were used as model inputs. To ensure data consistency, all features were normalized. Three machine learning models, SVM, KNN, and decision tree, were used to evaluate the generalization ability of these biomarkers. A 5-fold cross-validation strategy was used for model training and evaluation, ensuring consistent representation of each disease category.

### Histopathological assessment

Upon completion of all experimental protocols, rabbits were euthanized via intravenous overdose of sodium pentobarbital. Histopathological assessment was performed on enucleated eyes using hematoxylin–eosin-stained slides.

### Statistical analysis

The Pearson correlation coefficient between biomarkers was calculated to measure the relationship between these biomarkers. Data normality was assessed using the Shapiro–Wilk test, with a significance threshold of *P* > 0.05 indicating adherence to a normal distribution. In the animal model dataset, paired *t* tests were applied to evaluate longitudinal changes in the experimental and control groups for biomarkers that met the normality assumption. Nonnormally distributed biomarkers were analyzed using Wilcoxon signed-rank tests. To correct for multiple comparisons, the Holm–Šidák method was performed to maintain a family-wise error rate of *α* = 0.05. A *P* value <0.05 was considered statistically significant. In the clinical lymph node model, one-way ANOVA was performed, with Tukey’s test for multiple comparisons. A *P* value <0.05 was considered statistically significant. All analyses were performed using GraphPad Prism 10 (GraphPad Software, San Diego, CA, USA) and MATLAB 2024b (MATLAB, MathWorks, Natick, MA, USA).

## Ethical Approval

All animal experimental procedures were reviewed and approved by the animal care committee of ShanghaiTech University (Protocol #20251118001). The clinical patient studies were approved by the ethics committee of Peking University Third Hospital (Protocol #2024-795).

## Data Availability

The authors declare that the main data supporting the results in this study are available within the paper and its supplementary materials. The in vivo data can be made available from the corresponding author upon reasonable request and with permission for academic purposes. The ULM reconstruction and AI codes are available for research purposes from the corresponding author upon reasonable request.
