# Supplementary material for: A General-Purpose Framework for Microvascular Reconstruction and Quantitative Analysis in Ultrasound Localization Microscopy
Source: BME Front. 2026 Aug 3;7:0295. doi: 10.34133/bmef.0295 (PMC13429912; doi:10.34133/bmef.0295)
Supplement: Supplementary 1 — Figs. S1 to S7 Table S1 [file bmef.0295.f1.docx]

**Supplementary Online Content**


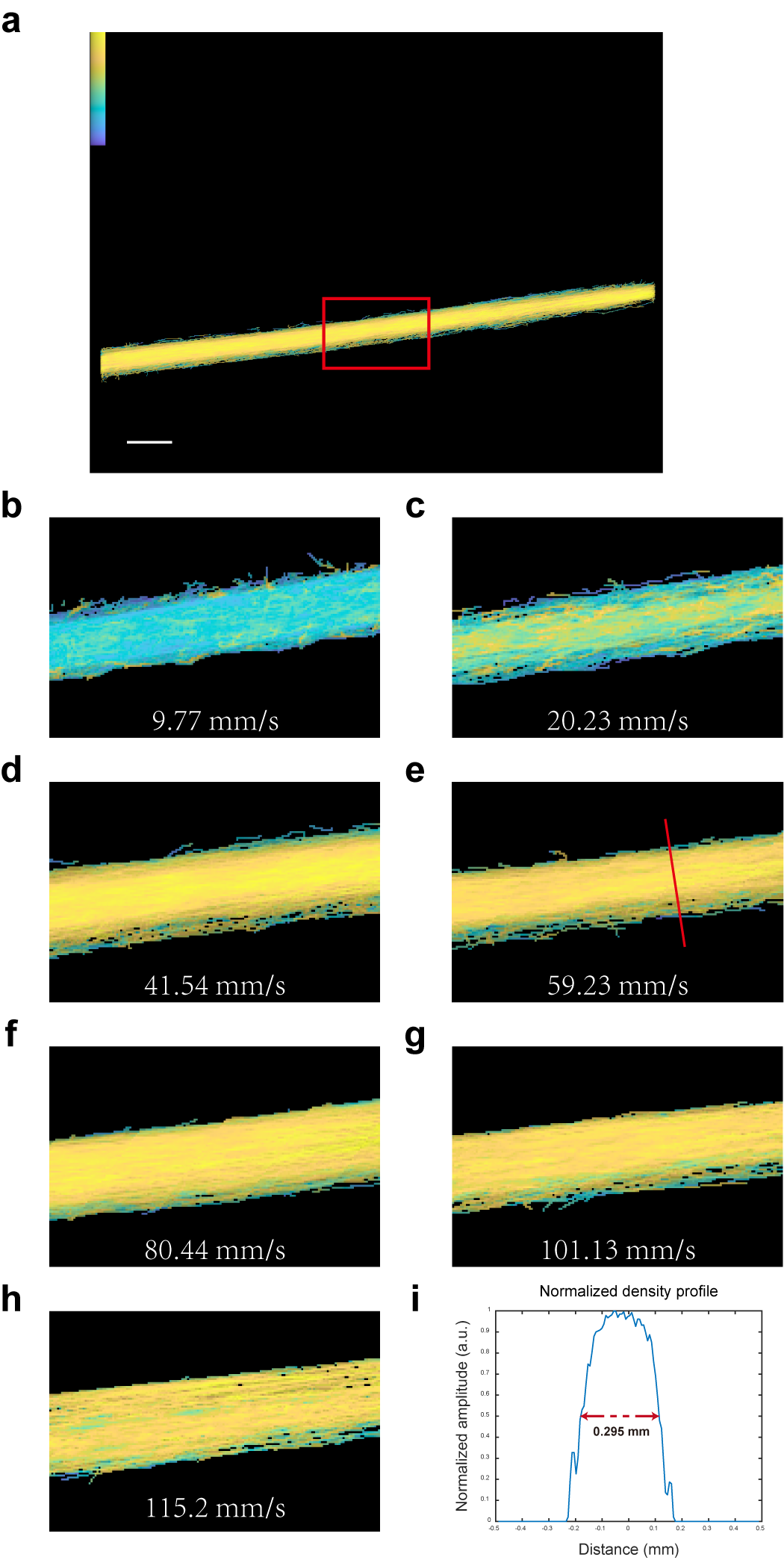


**Supplementary Fig. 1. Reconstructed ULM velocity images of the flow phantom.** (a) Schematic diagram of the entire reconstructed flow phantom. The red box represents the extracted ROI. (b-h) Reconstructed ROI images and measured velocities at 10 mm/s, 20 mm/s, 40 mm/s, 60 mm/s, 80 mm/s, 100 mm/s, and 120 mm/s. The red line represents the extracted cross section. (i) The blue line is the normalized density profile, and the red dashed line is the full-width at half-maximum (FWHM) of the profile. Scale bar: 1 mm.


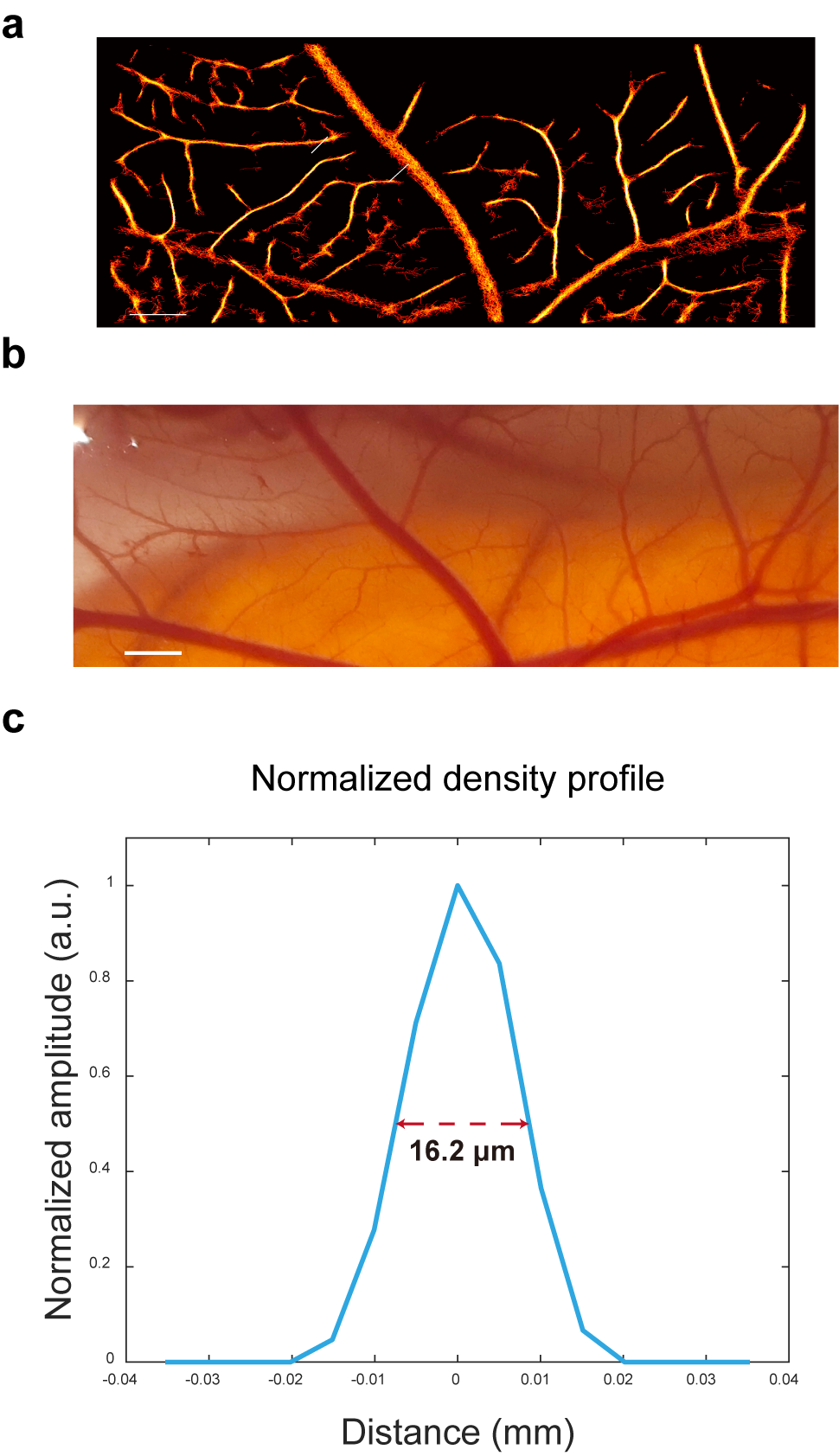


**Supplementary Fig. 2. CAM images.** (a) Reconstructed super-resolution blood flow density map. White lines represent extracted cross sections. (b) Optical contrast image (c) The blue line is the normalized density profile and the red dashed line is the FWHM of the profile. Scale bar: 1 mm.


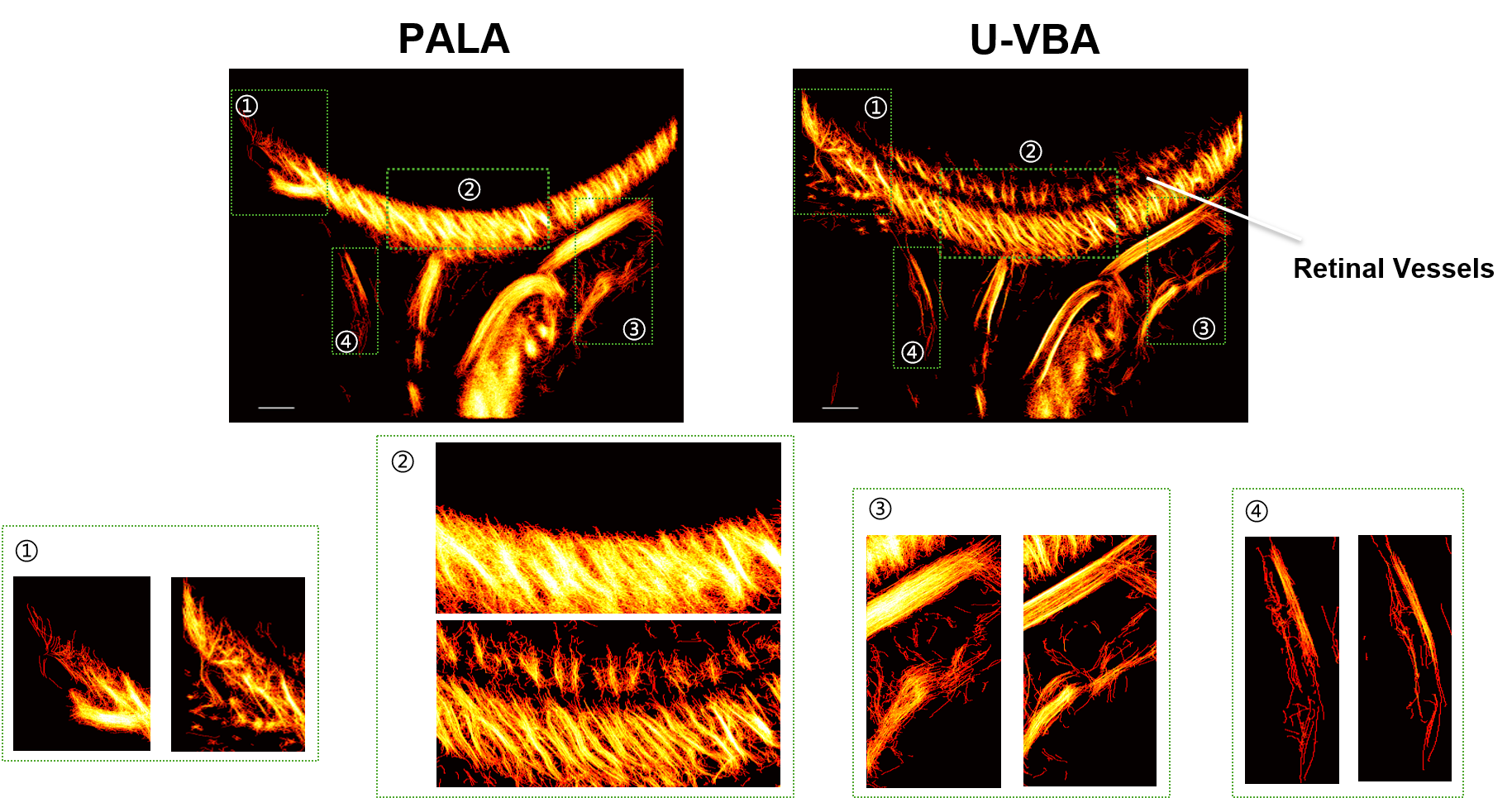


**Supplementary Fig. 3. Comparative analysis of microvascular reconstruction by PALA** **(limit of 200 bubbles per frame) and the proposed U-VBA framework.** Representative vascular density maps of the rabbit eye reconstructed by the open-source PALA algorithm (Left) and our U-VBA (Right) from the same dataset. Magnified views of four corresponding regions (green boxes) are shown beneath. Scale bar: 1 mm.


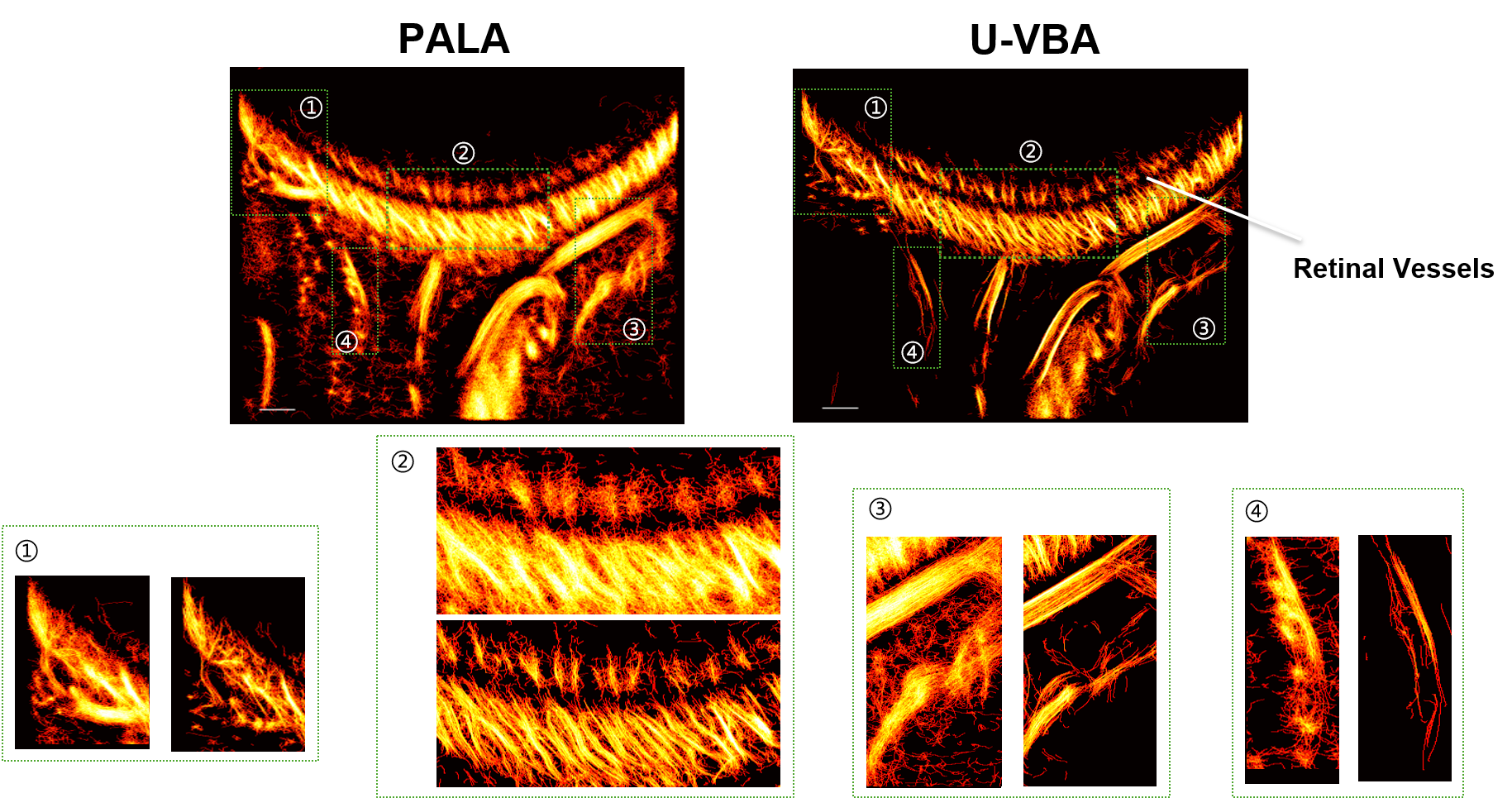


**Supplementary Fig. 4. Comparative analysis of microvascular reconstruction by PALA (no limit) and the proposed U-VBA framework.** Representative vascular density maps of the rabbit eye reconstructed by the open-source PALA algorithm (Left) and our U-VBA (Right) from the same dataset. Magnified views of four corresponding regions (green boxes) are shown beneath. Scale bar: 1 mm.


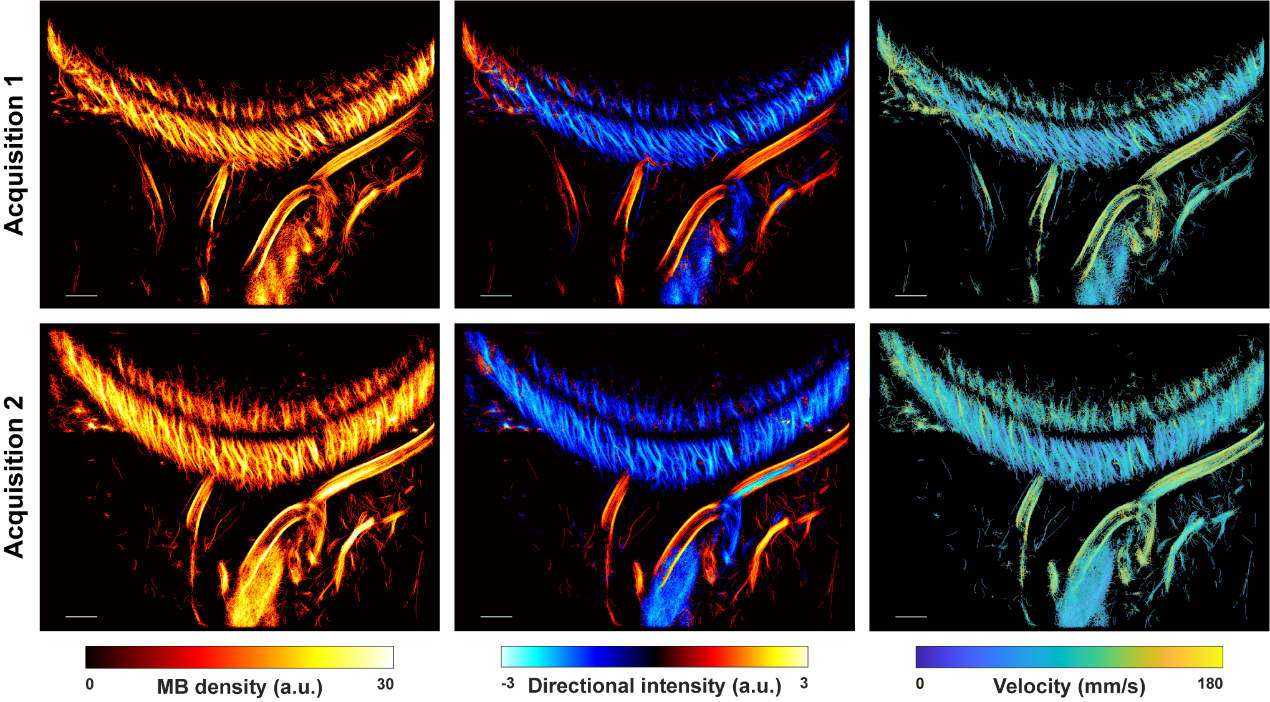


**Supplementary Fig. 5. Repeatability assessment in a single rabbit eye.** Two independent acquisitions of the same eye were performed at different time points under identical imaging parameters. Columns (left to right) show the vascular density map, directional vascular density map, and flow velocity map. Rows (top to bottom) correspond to Acquisition 1 and Acquisition 2. Scale bar: 1 mm.


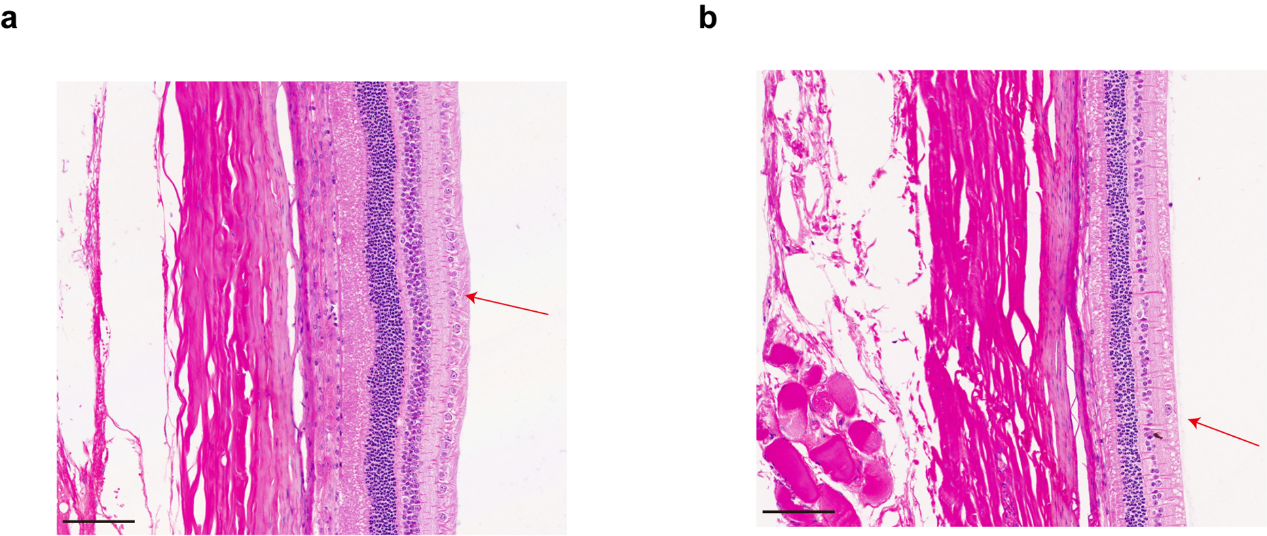


**Supplementary Fig. 7. Histological assessment of the rabbit retina under normal and optic nerve injury conditions.** (a) Hematoxylin and eosin (HE) staining of a control eye. (b) HE staining results of experimental eyes. The red arrows point to the retinal ganglion cells. Scale bar: 1 mm.


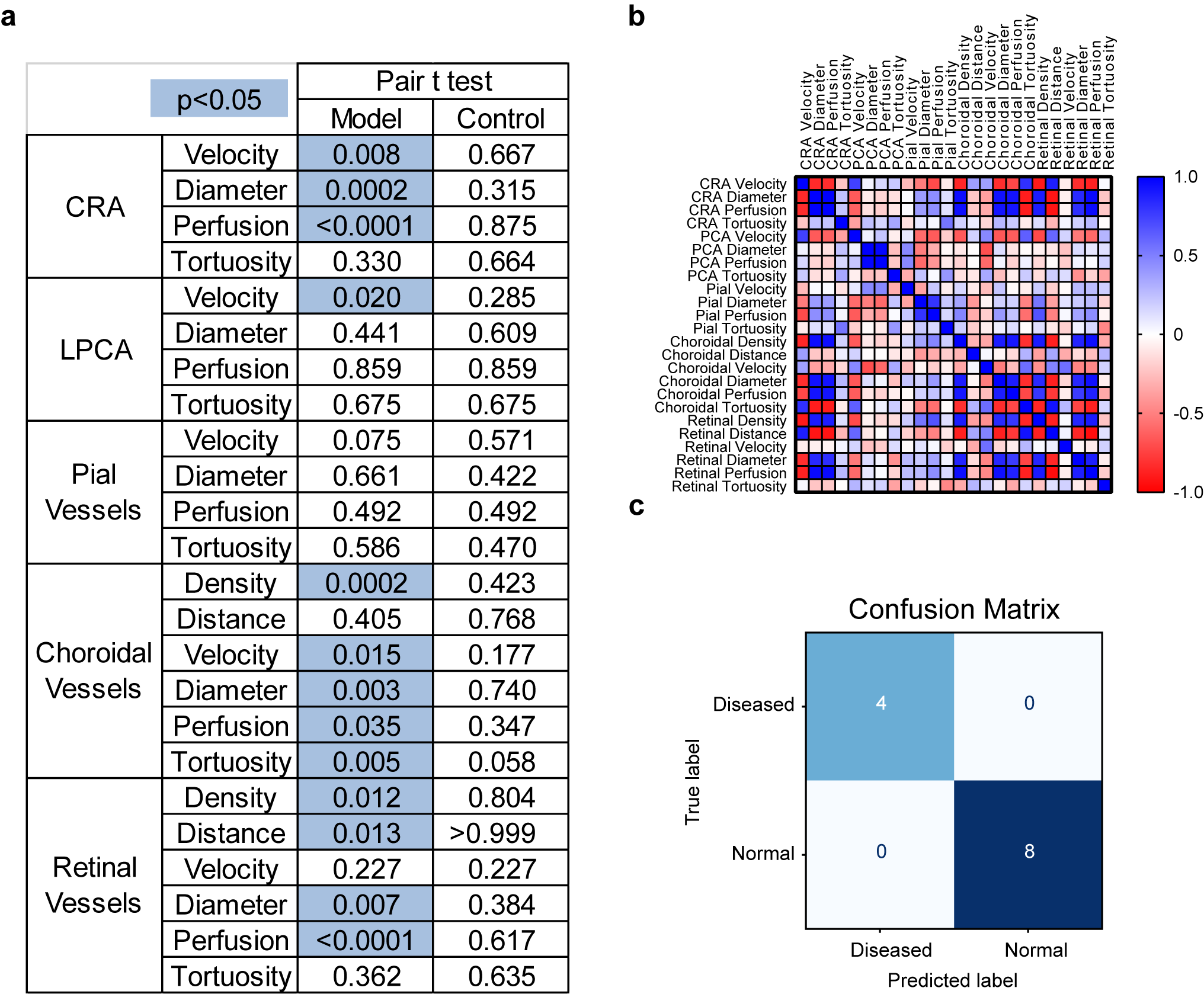


**Supplementary Fig. 6. Reliability analysis of biomarkers in diagnosing lesions.** (a) Results of paired t-test and Holm's multiple test correction. (b) Confusion matrix was generated for the trained diagnostic model. (c) Correlation matrix of all biomarkers (Pearson correlation coefficient r).

|  | Acquisition 1 | Acquisition 2 | Relative difference |
| --- | --- | --- | --- |
| Choroidal Density | 0.84 | 0.85 | 1.183% |
| Retinal Density | 0.5 | 0.5 | 0.000% |
| CRA Velocity | 90.21 | 89.29 | 1.025% |
| CRA Diameter | 0.55 | 0.543 | 1.281% |
| CRA Perfusion | 21.432 | 20.677 | 3.586% |
| PCA Velocity | 91.87 | 93.02 | 1.244% |
| PCA Diameter | 0.566 | 0.557 | 1.603% |
| PCA Perfusion | 23.115 | 22.666 | 1.962% |
| Pial Velocity | 55.41 | 52.948 | 4.544% |
| Pial Diameter | 0.1 | 0.1065 | 6.295% |
| Pial Perfusion | 0.45 | 0.472 | 4.772% |
| Choroidal Velocity | 50.81 | 53.73 | 5.586% |
| Choroidal Diameter | 0.106 | 0.109 | 2.791% |
| Choroidal Perfusion | 0.708 | 0.639 | 10.245% |
| Retinal Velocity | 38.969 | 38.219 | 1.943% |
| Retinal Diameter | 0.083 | 0.0825 | 0.604% |
| Retinal_Perfusion | 0.442 | 0.476 | 7.407% |
| CRA Tortuosity | 43.237 | 44.959 | 3.905% |
| PCA Tortuosity | 39.06 | 40.008 | 2.398% |
| Pial Tortuosity | 32.336 | 32.418 | 0.253% |
| Choroidal Tortuosity | 32.791 | 31.814 | 3.025% |
| Retinal Tortuosity | 31.063 | 31.532 | 1.499% |
| Retinal Distance | 0.25 | 0.23 | 8.333% |
| Choroidal Distance | 0.27 | 0.29 | 7.143% |
| Average difference |  |  | 3.443% |

**Supplementary Table 1 | Test-retest reliability of U-VBA-derived vascular biomarkers.** Biomarkers were extracted from two repeated acquisitions of the same eye in the same rabbit, acquired two days apart under consistent anesthesia and positioning protocols. The relative difference was calculated as | Acquisition 2 - Acquisition 1| / [(Acquisition 2 + Acquisition 1)/2] × 100%.
